# Sexual and injection-related risks in Puerto Rican-born injection drug users living in New York City: A mixed-methods analysis

**DOI:** 10.1186/1477-7517-8-28

**Published:** 2011-10-17

**Authors:** Camila Gelpí-Acosta, Holly Hagan, Samuel M Jenness, Travis Wendel, Alan Neaigus

**Affiliations:** 1 National Development and Research Institutes, Inc., New York, NY, USA; 2 New York University, College of Nursing, New York, NY, USA; 3 Department of Epidemiology, University of Washington, Seattle, USA; 4 John Jay College of Criminal Justice, City University of New York; 5 New York City Department of Health and Mental Hygiene, New York, NY, USA

## Abstract

**Background:**

These data were collected as part of the National HIV Behavioral Surveillance (NHBS) study. NHBS is a cross-sectional study to investigate HIV behavioral risks among core risk groups in 21 U.S. cities with the highest HIV/AIDS prevalence. This analysis examines data from the NHBS data collection cycle with IDU conducted in New York City in 2009. We explored how the recency of migration from Puerto Rico (PR) to New York City (NYC) impacts both syringe sharing and unprotected sex among injection drug users (IDU) currently living in NYC.

**Methods:**

We used a mixed-methods approach to examine differences in risk between US-born IDU, PR IDU who migrated to NYC more than three years ago (non-recent migrants), and PR IDU who migrated in the last three years (recent migrants). Respondent-driven sampling (RDS) was used to recruit the sample (n = 514). In addition, qualitative individual and group interviews with recent PR migrants (n = 12) and community experts (n = 2) allowed for an in-depth exploration of the IDU migration process and the material and cultural factors behind continued risk behaviors in NYC.

**Results:**

In multiple logistic regression controlling for confounding factors, recent migrants were significantly more likely to report unprotected sexual intercourse with casual or exchange partners (adjusted odds ratio [AOR]: 2.81; 95% confidence intervals [CI]: 1.37-5.76) and receptive syringe sharing (AOR = 2.44; 95% CI: 1.20-4.97) in the past year, compared to US-born IDU. HIV and HCV seroprevalence were highest among non-recent migrants. Qualitative results showed that risky injection practices are partly based on cultural norms acquired while injecting drugs in Puerto Rico. These same results also illustrate how homelessness influences risky sexual practices.

**Conclusions:**

Poor material conditions (especially homelessness) may be key in triggering risky sexual practices. Cultural norms (ingrained while using drugs in PR) around injection drug use are perpetuated in their new setting following an almost natural flow. These norms may have a particular stronghold over risky drug injection practices. These results indicate that culturally appropriate HIV and HCV prevention and education services are needed. In addition, homelessness should be addressed to reduce risky sexual practices.

## Background

New York City (NYC) is a destination point for immigrants from around the world. As of 2000, 44% of its adult population was born outside the United States, with 30% of foreign-born adults reporting a Hispanic/Latino ancestry [1]. Injection drug users (IDU) in NYC are similarly diverse. Recent studies have estimated that approximately half of NYC IDU are Hispanic [2,3], and that many within that group are Puerto Rican-born IDU (PR IDU) [4-6].

For PR IDU, NYC-bound migration is triggered by many factors, such as moving with family members, seeking employment or drug treatment, and evading law enforcement [4,7]. Despite a large population of people living with HIV/AIDS in Puerto Rico (over 35,000; among whom injection drug use continues to be the primary transmission source), and the second-highest rate of HIV infection among U.S. states and territories [8], there are currently only six methadone programs, seven buprenorphine treatment programs [9], and eight syringe exchange programs (SEP) in operation in Puerto Rico. Many of these programs are concentrated in the San Juan metropolitan area. Several of the SEP are faith-based, as religion plays a central role in treatment paradigms among healthcare professionals and in governmental health policies [10,11].

Recent research has shown how environments and social structures influence injection drug use behaviors [12-16]. Poverty, law enforcement, drug policies, homelessness, drug treatment and SEP coverage are among the social factors that influence IDU risk behaviors. Also, racial discrimination and marginalization have also been identified as critical considerations when studying risk-taking behaviors among destitute drug users and their communities [17]. In addition, sociologists have explored how culture, generally defined here as a pliable system of norms, values, beliefs and practices that are unified by language, geography and a common history, is an intrinsic part of the social structures that govern individual and group behavior [18-20]. Regarding PR migrant IDU, researchers have identified important cultural markers (i.e., heritage, traditions, Latino/Hispanic identity and a sense of belonging to a community) that differentiate this population from other IDU in the U.S. [21,22].

All individuals in society develop within specific cultural settings, and IDU are no exception. Because of specific social and structural conditions coinciding in the world of illicit drug use (e.g., criminalization, exposure to police, stigma, fear, violence and marginalization) particular cultural norms develop among drug users [18-20]. Group solidarities and norms emerge to deal with the pressures exerted by drug policies, law enforcement agents and drug use craving and/or withdrawal symptoms. All these factors underlie in varying degrees risk behaviors among IDU. The unavailability of drug treatment and SEP services may also trigger individual and group norms. Among PR migrant IDU, every day practices stem, at least partly, from the place-specific logics where injection drug use was initiated.

In order to operationalize how these structures influence drug users' lives and risk behaviors, Pierre Bourdieu's concept of 'habitus' is useful. Habitus refers to the manifestation of a process in which social structures (such as culture) are embodied and reproduced (unconsciously) by groups and individuals [23,24]. It is the set of conceptual "gridlines" through which individuals understand their world and move within it, almost as if their perceptions and actions were "second nature". This concept has been used by drug researchers to demonstrate the ways in which social structures, such as extreme socioeconomic and racial marginalization, manifest in drug users' practices [25]. Similarly, our objective is to show how *place-*specific cultural norms acquired while injecting drugs in Puerto Rico, and at times unconsciously, continue to inform continued individual and collective risk behaviors among this population in NYC. This habitus migration may help explain IDU continued injection and sexual risk in spite of increased access to SEP in their new setting.

Previous research showed that PR IDU bring cultural norms of syringe sharing with them to NYC since most PR IDU in NYC started injecting drugs in Puerto Rico prior to migrating; and this was associated with higher levels of syringe sharing in NYC [4]. Other IDU migration studies have also discussed the interactions between old and new drug injection settings on migrant IDU risk behaviors [26,27]. Often, new settings bring along new rules and degrees of access to sterile injection equipment. The ways in which these vulnerable populations assimilate or reject these structural changes are not fully understood. In this paper, we examine how the previous drug-injection settings of PR IDU continue to inform their risk behaviors in NYC. Moreover, our study expands scientific knowledge on this population by outlining how and why continued risk behaviors are reproduced in their new setting. In addition, we will describe some of these PR-specific cultural norms and how they manifest in a group of recent PR migrants in NYC.

Despite the wider availability of drug treatment, syringe exchange, and other services in NYC motivating migration from Puerto Rico to NYC, many PR IDU do not use these programs, and of those who do, many cannot easily eschew the risky cultural norms of their past [6]. PR IDU in NYC have experienced high levels of homelessness and poverty, which may trigger sexual risk in partnerships in which sex is exchanged for money or drugs [28]. Disarrayed material conditions in NYC, along with shared cultural markers (i.e., monolingual Spanish, heritage, Latino/Hispanic identity, etc), may trigger group solidarity and further perpetuate their PR-specific norms in the new setting. These migration aspects and the background HIV risk and prevalence in Puerto Rico can potentially impact the scope of HIV infection among IDU living in NYC: 16% of NYC HIV cases in 2007-8 attributed to injection drug use were among PR IDU [2].

In this analysis, we explore how recent migration from PR to NYC impacts both syringe sharing and unprotected sex among NYC IDU. While a previous study on PR IDU migrants examined a similar time variable (recent visits to NYC) [4], we defined it differently (fewer than 3 years living in NYC). Thus, we examine risk-behaviors among those PR IDU who have moved their residence to NYC. This approach allowed us to acquire more insight on the rationales behind residential moves and to identify the differences in continued risk-taking behaviors when compared to other subgroups in the sample.

## Methods

We used a mixed-methods approach to examine differences in risk between US-born IDU, PR IDU who migrated to NYC more than three years ago (non-recent migrants), and PR IDU who migrated in the last three years (recent migrants). Qualitative individual and group interviews with recent PR migrants and community experts allowed for an in-depth exploration of the IDU migration process and the material and cultural factors behind continued risk behaviors in NYC.

### Sampling

These data were collected as part of the National HIV Behavioral Surveillance (NHBS) study, described in detail elsewhere [29]. NHBS is a cross-sectional study to investigate HIV behavioral risks among core risk groups in 21 U.S. cities with the highest HIV/AIDS prevalence. This analysis examines data from the NHBS data collection cycle with IDU conducted in New York City in 2009.

### Procedures

Prior to the main data collection phase, from March to May of 2009, we conducted formative ethnographic research. One of the objectives of this ethnography was to get acquainted with the current NYC IDU population characteristics, including HIV related risk behaviors, in order to guide data collection. Ethnography involved informal non-recorded interviews, focus groups, individual key informants' interviews, field observations, and analysis of qualitative data. Our study ethnographer identified and recruited recent PR migrant IDU through street intercepts with Puerto Rican IDU in the Bronx and by interviewing recent PR migrant IDU researchers. In this analysis, we included findings from the focus groups and key informant interviews conducted with recent PR migrants and recent PR migrant IDU community researchers. Relevant topics included the migration process, including programs in Puerto Rico and in NYC involved in the process, reasons behind sexual and drug injection risk behaviors, and perceptions of HIV and HCV risk. Thorough notes during this process were taken and we analyzed the qualitative data guided by their relevance to these topics. All participants gave informed consent and received an incentive for their participation in both stages of the study.

In the main data collection phase, respondent-driven sampling (RDS) was used to recruit active drug injectors in NYC [30]. RDS requires recruitment by members of the target population who are socially linked. Study ethnographers recruited a small group of initial participants (called "seeds") who completed the study and then referred three other IDU. Seeds were recruited in areas of NYC where IDU are known to reside and highly active illicit drug markets thrive. One recent migrant PR IDU seed was selected to increase the odds for recent migrants' networks inclusion in the main survey. Participants referred by the seeds then completed the study and were also provided with three coupons for IDU peers they could refer to the study. Successive waves were recruited until the desired sample size was reached. Eligible IDU had to be 18 years or older, be proficient in English or Spanish, have injected illicit drugs at least once in the past 12 months, and reside in the NYC metropolitan area.

Trained interviewers administered a structured questionnaire with each recruit. The survey asked about sociodemographics, drug use and sexual behaviors, drug treatment participation, and HIV and hepatitis C (HCV) testing experiences. In addition, phlebotomists collected blood specimens using venipuncture. Specimens were tested for HIV antibody on HIV1/2 enzyme-linked immunosorbent assay (ELISA) and HIV1 western blot platforms (Bio-Rad Laboratories, Hercules, CA) and HCV antibody on an ELISA platform (Abbott Laboratories, Chicago, IL). Individuals were paid incentives for completing the questionnaire, HIV/HCV testing, and peer recruitment. All study procedures were approved by the Institutional Review Boards of the participating organizations.

### Variables and Analyses

Participants were categorized into three groups based on their migration history: 1) US-born IDU (including those with and without PR ancestry); 2) IDU who migrated from Puerto Rico over three years ago; and 3) IDU who migrated from Puerto Rico within the last three years. This 3-year cut off was consistent with previous literature on risk among PR IDU coming to NYC [4], but Deren et al. referred to any type of travel between the two locations, while this analysis defines migration as a change of domicile. Participants who immigrated to the US from other countries were excluded from this analysis because it was inappropriate to include them with any of the three groups above.

We investigated two main outcome measures reviewed in this analysis: 1) receptive syringe sharing; and 2) unprotected casual/exchange sex. The first is defined as injecting drugs in the past year with a syringe that someone else has already used. The second is defined as past-year unprotected vaginal or anal sex with non-main partners, or partners with whom sex is traded for things like money or drugs. Three main sociodemographic covariates included were: 1) poverty, defined as having a 2008 income below the Federal poverty line; 2) homelessness (living on the street, in a shelter, or a single room occupancy apartment) in the past 12 months; and 3) incarceration in a prison or jail for at least one day in the past 12 months. Additionally, we categorized anyone below 30 years of age as a young IDU.

Data analysis examined differences in sociodemographics, sexual and injection-related risks, and disease outcomes between the three groups. All analyses were weighted using the Respondent-Driven Sampling Analysis Tool (RDSAT) (Cornell University, Ithaca, NY), which adjusts for recruitment bias in peer-referral sampling [30]. Multivariate logistic regression models were created to model the association between the three-level PR migration exposure variable and the two behavioral risk outcomes. Covariates included in the models met data-based criteria for confounding: when entered in the model, the coefficient for the main predictor variable (PR migration) changed by more than 10% [31].

## Results

### Quantitative Results

A total of 514 non-seed IDU were eligible and completed the NYC NHBS study, of whom 26 were foreign-born IDU removed from this analysis, leaving a final analytic sample of 488. As Table 1 shows, the sample was 79% male and 21% female. Fifty-percent were Hispanic (all Puerto Rican IDU in the sample -from the US and from PR- fall within this category), 37% White and 13% Black. The mean age was 40. Two-thirds earned less than $10,000, 62% were homeless, and one-third had been incarcerated in the past year. Two-thirds had unprotected vaginal or anal sex with a heterosexual partner and 22% engaged in this with a casual or exchange partner. Forty-five percent reported binge alcohol use and 66% reported noninjection drug use, with many of those using crack (36%). In terms of risky injection behaviors, 28% reported receptive syringe sharing and 41% shared other injection supplies (cookers, water and cottons) in the past year. Overall, 17% tested positive for HIV and 72% tested positive for HCV.

**Table 1:** Sociodemographics, Sexual Risk Factors, Drug Use & Risk, and Disease Outcomes by Puerto Rican Immigration Status, among New York City Injection Drug Users, 2009, n = 488

		Immigration Status			
	Total	US-Born	PR- Immigrated > 3 Years Ago	PR-Immigrated ≤ 3 Years Ago	
	**%**	**%**	**%**	**%**	**P**

**Gender**					0.09
Male	78.8	76.2	90.5	74.5	
Female	20.9	23.4	9.5	25.5	
Transgender	0.3	0.4	0.0	0.0	
**Race/Ethnicity**					< 0.01
Black	12.8	21.5	0.0	0.0	
Hispanic	50.2	35.1	100.0	100.0	
White	36.7	42.9	0.0	0.0	
Other	0.3	0.5	0.0	0.0	
**Age**					0.03
18-29	11.2	11.7	2.4	13.0	
30-39	27.8	24.8	35.1	37.8	
40-49	43.2	44.9	39.8	44.1	
50 +	17.8	18.5	22.7	5.1	
**Sociodemographics**^**1**^					
Homeless	62.1	59.6	59.3	85.2	0.01
Income < $10,000	65.0	61.4	72.1	87.1	< 0.01
Incarcerated	33.3	36.0	28.3	25.3	0.23
**Sexual Risk Factors**^**1**^					
Unprotected Intercourse	64.3	60.7	72.0	81.9	0.01
UI with Casual/Exchange Partner	21.5	17.2	32.7	37.4	< 0.01
≥ 3 Total Partners	23.0	20.0	32.8	28.7	0.03
Mean (Median) Total Partners	3.6 (1)	4.0 (1)	2.3 (1)	3.3 (2)	0.07
**Alcohol/Non-Injection Drug Use**^**1**^					
Binge Alcohol Use	44.9	46.4	36.7	37.6	0.22
Binge Alcohol Use ≥ 1x/week	25.1	25.9	19.7	23.9	0.53
NI Drug Use	65.5	71.4	53.3	36.9	< 0.01
NI Drug Use ≥ 1x/week	48.8	53.2	37.1	35.6	< 0.01
NI Crack Use	36.3	40.8	24.4	21.8	< 0.01
**Injection Drug Use**^**1**^					
Drug Injection ≥ 1x/day	83.4	80.7	89.0	98.8	< 0.01
Drugs Injected					
Heroin Alone	89.8	93.8	73.5	87.8	< 0.01
Speedballs	55.5	51.3	71.6	72.8	< 0.01
Cocaine Alone	43.3	48.4	32.0	23.2	< 0.01
Receptive Syringe Sharing	27.6	24.4	30.4	40.5	0.08
Mean (Median) RSS Partners	0.9 (0)	0.8 (0)	1.0 (0)	1.5 (0)	0.04
Cooker, Cotton, Water Sharing	41.2	40.0	38.8	52.5	0.34
**Disease Outcomes**					
HIV Seroinfection (n = 485)	16.5	13.6	31.2	7.0	< 0.01
HCV Seroinfection (n = 478)	72.0	69.4	89.0	77.0	< 0.01

^1 ^Timeframe: in the past 12 months

By migration category, 72% of participants were US-born (36% of whom had PR ancestry), 18% were non-recent PR migrants, and 10% were recent PR migrants. Recent migrants were more likely to be younger (p = 0.03), homeless (p = 0.01), and living in poverty (p < 0.01) in the past year. Recent migrants had significantly higher levels of unprotected sexual intercourse overall (p = 0.01), and specifically of unprotected sexual intercourse with casual/exchange partners (37% vs. 33% for non-recent migrants and 17% for US born, p < 0.01). Noninjection drug use overall (p < 0.01) and specifically noninjection crack use (p < 0.01), was significantly lower among recent migrants. For injection risks, recent migrants were significantly more likely to inject at least daily (p < 0.01) and inject speedball (p < 0.01). With marginal significance, recent migrants were more likely to share syringes (p = 0.08), with a significantly higher number of median sharing partners (p = 0.04). Finally, HIV (31.2%) and HCV (89%) seroprevalence were highest among non-recent PR migrants.

In a subanalysis of recent PR migrants (data not shown), 98% started injecting drugs while still in Puerto Rico (compared with 69% of the non-recent PR migrants). In addition, 67% of recent migrants reported that they moved to NYC to access drug treatment services, compared with 46% of non-recent migrants. Seventy-nine percent were monolingual Spanish speakers.

Table 2 presents factors associated with past-year unprotected sex with a casual/exchange partner and receptive syringe sharing. In bivariate analysis, female IDU, black IDU, and older IDU were all less likely to report unprotected sex with a casual/exchange partner. IDU who were incarcerated in the past year, those who engaged in binge alcohol use, and PR migrants (both recent and non-recent) were all significantly more likely to report this sexual risk. In multiple logistic regression controlling for confounding factors (age and incarceration), both recent migrants (AOR = 2.81; 95% CI = 1.4-5.8) and non-recent migrants (AOR = 2.86; 95% CI = 1.6-5.0) were significantly more likely than US-born IDU to engage in unprotected sex with a casual/exchange partner.

**Table 2:** Factors Associated with Past Year Unprotected Sex with a Casual/Exchange Partner and Past Year Receptive Syringe Sharing, among New York City Injection Drug Users, 2009, n = 488

	**Unprotected Sex with Cas/Exch Partner**^**1**^					**Receptive Syringe Sharing**^**1**^				
	
	%	OR	95% CI	AOR	95% CI	%	OR	95% CI	AOR	95% CI
**Overall**	21.5	-	-			27.6	-	-		
**Gender**										
Male	24.0	1.00				23.6	1.00			
Female	12.7	0.46	0.25-0.86			41.8	2.32	1.48-3.63		
Transgender	-	-	-			-	-	-		
**Race**										
Black	5.3	1.00				16.2	1.00			
Hispanic	27.4	6.76	2.47-18.50			30.1	2.23	1.17-4.25		
White	20.7	4.66	1.65-13.19			29.6	2.18	1.11-4.29		
Other	-	-	-			-	-	-		
**Age**										
18-29	37.3	3.33	1.49-7.43			41.7	6.48	2.68-15.65		
30-39	31.8	2.61	1.33-5.13			37.5	5.42	2.51-11.73		
40-49	13.9	0.90	0.45-1.79			25.1	3.04	1.43-6.47		
50+	15.2	1.00				9.9	1.00			
Continuous	-	0.94	0.92-0.96	0.94	0.92-0.97	-	0.96	0.94-0.98	0.94	0.92-0.96
**Puerto Rican Immigration**										
U.S. Born	17.3	1.00		1.00		25.2	1.00		1.00	
PR Immigrated > 3 Years	33.0	2.35	1.37-4.02	2.85	1.61-5.03	31.3	1.35	0.80-2.30	1.86	1.04-3.31
PR Immigrated ≤ 3 Years	37.7	2.89	1.47-5.69	2.81	1.37-5.76	41.6	2.12	1.10-4.07	2.44	1.20-4.97
**Sociodemographics**^**1**^										
Homeless	23.6	1.41	0.90-2.21			30.4	1.47	0.97-2.22		
Income > $10,000	23.1	1.15	0.74-1.79			23.9	0.75	0.49-1.14		
Incarcerated	29.9	2.06	1.33-3.18	1.89	1.19-3.02	27.1	0.97	0.64-1.46		
**Substance Use**^**1**^										
NI Crack Use	19.1	0.80	0.51-1.25			41.9	3.04	2.03-4.55	3.01	1.95-4.65
Binge Alcohol Use	28.3	2.05	1.33-3.16			34.1	1.79	1.21-2.65		
**Injection Drug Use**^**1**^										
Injection ≥1x/day	21.6	1.04	0.58-1.85			27.5	0.98	0.58-1.66		

^1 ^Timeframe is in the past 12 months

In bivariate analysis, receptive syringe sharing was significantly more likely among female, White or Hispanic, and younger IDU. Syringe sharing was also significantly higher among noninjection crack users and recent PR migrants. In multiple logistic regression controlling for confounding factors (age and noninjection crack use), both recent migrants (AOR = 2.44; 95% CI = 1.2-5.0) and non-recent migrants (AOR = 1.86; 95% CI = 1.04-3.31) were significantly more likely than US-born IDU to share syringes. Noninjection crack use was also significantly associated with syringe sharing (AOR = 3.01; 95% CI = 2.0-4.7).

### Qualitative Results

In qualitative ethnographic research, 61 participants were interviewed in 6 focus groups (8 participants per focus group), 11 individual community key informants (IDU) and 2 key informants (community experts). Of the 61, 12 were recent PR migrants included in this analysis. Eight of these were part of a focus group held with recent PR migrants and 4 more were individually interviewed. At the time of the ethnographic research, most were homeless and living on the street, while others were living in transitional housing institutions (so-called "three-quarter houses"). All were males aged 20 to 43 years old and living in the Bronx. Most participants knew each other, but had met for the first time in NYC. All were monolingual Spanish speakers who had migrated to NYC through faith-based drug treatment programs. All qualitative data collection was carried out in Spanish. All 12 were also recruited into the main survey.

### Migration process

Migration was the first and most heated topic in the focus group. Anger and frustration were palpable in their narratives of moving to the US to attend drug treatment programs. They explained that mayors of several municipalities in Puerto Rico, special police programs and many Pentecostal ministers assist IDU families (and individuals) financially to enroll PR IDU in "drug treatment programs" in NYC. One participant also mentioned that staff at correctional facilities in Puerto Rico sometimes assists the IDU migration process. Other major cities of the US Eastern seaboard were also mentioned as migration destinations for many PR IDU (including Boston and Philadelphia). Once in NYC, many reported being picked up at the airport by Pentecostal ministers or by their church staff.

While in Puerto Rico, they were not made aware that the programs they were volunteering to join were faith-based. One key informant explained, "Before migrating, I was offered drug treatment and a job, a chance to get out of trouble. That's why I came here." Upon arrival, many found themselves enrolled in programs that did not fulfill these expectations. They explained that these programs are a "scam." They complained about the conditions of these facilities and the religious focus of the programs, including "mandated morning praying routines," "bedbugs," "sleeping on church floors," "overcrowding," "the abstinence-only model," and "charging their Medicaid cards for services they never receive." All of the participants had dropped out of these programs by the time of the interview. In fact, most participants reported dropping out of these programs within 3 months of enrollment. Because housing was offered as part of treatment, homelessness followed.

### Reasons behind risky sexual behaviors

Most participants were very open about their sexual risks and drug use. Among other things, heterosexual risk was explained in terms of recurring monetary needs (usually to get drugs), getting temporary shelter, and unexpected sexual encounters while using drugs (especially speedball). While all participants admitted they rarely (if ever) used condoms while in Puerto Rico, they also view their current poor material conditions as limiting their ability to refrain from engaging in unprotected sex with casual/exchange partners.

In a key informant interview, a former Bronx-based syringe exchange program employee and a social psychologist who studies PR IDU migration to NYC said that "homeless IDU who have recently migrated from Puerto Rico find people in these programs [syringe exchange programs and other community based organizations] that have housing." Some recent PR IDU migrants who are homeless find themselves in a situation where they may have little choice but to engage in a potentially risky sexual situation in order to avoid (even if temporarily) homelessness. In the focus group, some explained sometimes this is the only way to get shelter.

While lack of condom use might be partly explained by the deeply rooted "macho" sexual identities characteristic of many Hispanic cultures, it is also related to precarious material circumstances that prevent them from using condoms. Sex work patrons often pay more for unprotected sex. Some also mentioned that "speedball" has a twofold effect: (1) it increases their desire to have sex, while (2) it constrains them from using condoms. They report condoms limit the desired sexual sensation already compromised by the pharmacological effects of the drug combination ("speedball"). Craving drugs, being high on drugs, lack of money and homelessness are some of the reasons for unprotected exchange/casual partnerships.

### Reasons behind risky injection

Participants also suggested that syringe sharing behaviors have different justifications, explaining that a certain "mentality" developed while injecting drugs in Puerto Rico. "Trust" is also one of the primary reasons for their current sharing of injection supplies. "These are my brothers here," one of the focus group participants asserted, "I'll do anything for them and I know they would do anything for me." For them, "brothers" ("hermanos") are those who also come from Puerto Rico, share the same drug-using norms practiced in Puerto Rico and are immersed in similar material circumstances (homelessness, "three-quarter house" transitional housing, and "faith-based" program drop-outs). The IDU-specific language normally used (i.e., "manteca" (literally, "lard", but here the most common slang term for heroin among this population), "droga" (literally, "drug" but exclusively signified as heroin by this population), "la cura" ("the cure" (for heroin withdrawal)) is another commonality that helps unify them as a group. They will give away their last sterile syringe to their peers in the same way they will share their syringes between them, or share drugs with a peer who is "sick". There is a clear familial bonding in this population. Their treatment of each other displays love, trust, and a deeply rooted connection.

Yet it also seemed that sharing injection supplies is "second nature" among these individuals, an unquestioned, and perhaps unconscious, habit. For instance, while discussing the dangers of injecting in the neck (i.e., hitting an artery could cause a stroke; hitting a nerve can be extremely painful), a focus group participant explained that "this is how I learned to do this", as he held his breath making the veins of his neck swell. Every day, he injects in the neck without any need for assistance, although this is generally considered by IDU to be a risky practice that usually is facilitated by another injector. This risk-taking behavior seemed to follow a natural flow. This participant, appearing almost as if unaware of the risks, continued "It's the best hit", while his peers' body language silently agreed. This is an example of what participants meant when they spoke of a certain "mentality".

For instance, after the ethnographer's questions around continued syringe sharing despite access to free and sterile needles, one recent PR migrant IDU who we interviewed individually as a community key informant explained,

Participant: Because that's the way of doing things in the street [in Puerto Rico]. Since there are no places to exchange syringes, then... that's how it is, you use it first and then I use it.

Interviewer: Even though you have access now? Is this some kind of rule that you bring to here with you?

Participant: "Over there the mentality is different. That's just the way it is. We could take 40 "ganchos" (literally, "pins"; here a slang term for syringes) on Friday, for Saturday and Sunday. But we don't. Nobody does. And then on Saturdays and Sundays they take them from over there, from the shooting [pointing at the "shooting gallery" [injection location] across the street from where we were sitting]. It's just the way it is.

Aside from this PR IDU-specific "mentality", he also mentioned that "being homeless" and feeling "lonely" [in the new setting] may trigger in some a sense of "carelessness", almost as if their lives cannot get any worse than it already has. He used the term "estorbo público" (a public nuisance) to refer to himself. After living in NYC for the past 3 years, he is yet to find structural stability, learn English and to change his PR IDU "mentality". He is 43 years old and runs what seems to be a "temporary" "shooting gallery" (where he also sleeps) located in an abandoned building in the South Bronx. He has a $200/day "speedball" habit that he supports by selling heroin and cocaine. Most (if not all) of his "shooting gallery" patrons and clients are also recently migrated PR IDU. We asked him about the overall makeup of his drug users' network, to which he replied "All injectors from Puerto Rico. These people are abusive over here. The hang-out scene is different here. In Puerto Rico, we didn't allow certain things. We had rules. Over here, a 'snitch' can cop and sell drugs. You don't see that over there." We asked him if that was the reason why he didn't hang out with other PR IDU born in the United States (and usually bilingual) to which he replied affirmatively.

Quasi-familial bonding develops quickly among migrant PR IDU in NYC, because there is a sense of threat to their drug user identity (and their safety) by other street drug users who are unfamiliar with the "Puerto Rican way". The fact that most are Spanish monolingual, homeless IDU converging in NYC allows for this array of signs (e.g. - homelessness, monolingual Spanish, IDU from PR, etc) to be read as family-like and involving bonds of "brotherhood"; trust emerges from this because their everyday struggles in their new setting are very similar.

A focus group participant confirmed part of what the above participant said about risk during weekends. For him, part of the problem is that he gives away his sterile syringes, "especially during the weekends, because nobody has any on them". He also explained that some of his IDU peers are staying in "three-quarter houses", where they cannot have syringes or they will be ejected and will face homelessness again. Other group participants mentioned police harassment around syringe exchange programs and being scared of "syringe arrests" as some of the reasons for not carrying extra syringes on them.

### Perceptions of HIV and HCV risks

Upon probing around the risks for HIV and HCV, some said they were "already HCV positive". Although they are "scared" of HIV, trust in their "brothers'" HIV-negative self-reports is apparent. Their trust in their peers, combined with the typical "you don't think of that when you're sick" (which in their case happens often), provide for a powerful mix of social forces that set the stage for continued syringe sharing within this group. Despite ample access to free and sterile injection supplies in NYC, sharing paraphernalia is mostly an action informed by habits, trust and material constraints. Although most met for the first time in NYC, they quickly developed trusting relationships based on shared island-specific drug culture norms, drug injection habits and shared current material conditions (e.g., homelessness and poverty). It is also possible that these "brotherhood" sentiments are a way for these individuals to recreate their own Puerto Rico in a new setting that has proven to be hostile and non-trusting.

## Discussion

Similar to other studies of PR migrant IDU in NYC [28], our analyses showed that PR-born migrant IDU were more likely than US-born IDU in NYC to report unprotected sex with a casual/exchange partner and receptive syringe sharing. A recent study on this population showed that IDU born and living in Puerto Rico engage in riskier drug injection behaviors when compared to their counterparts in Massachusetts [22]. The ARIBBA study, which compared Puerto Rican IDU risk behaviors in Bayamón, PR and in Harlem, NYC, demonstrated similar findings [4]. This same study also found that Puerto Rican IDU in NYC who regularly injected drugs in Puerto Rico prior to migrating to NYC are more likely to engage in risky injection behaviors in NYC than Puerto Rican IDU who started injecting in NYC.

Our study found that for receptive syringe sharing, the risk was greatest among recent migrants. Formative research showed that many of the recent PR IDU bring along with them drug-injection behavioral routines that are somehow perpetuated in their new setting. There is an array of socioeconomic and cultural factors that converge to make this situation possible. Recent PR IDU migrants in NYC continue to share a sense of what the drug users' world should be like (the "Puerto Rico way"), despite the fact that they are now in NYC. They also perpetuate a familiar drug-user vocabulary, and carry on similar drug-using behaviors that speak to their times using drugs in Puerto Rico, where access to injection supplies was not a part of their lives. These norms, perceptions and habits continue to be present in their everyday lives. Their practices appear to follow an almost unconscious disposition towards risky drug injection practices. In this population, risky behaviors often take place as if "naturally". This is particularly true for injection risk behaviors (i.e., injecting in the neck and sharing injection equipment). While this shared habitus may facilitate their bonding processes, their current sharing of certain socio-structural limitations (monolingual Spanish speakers, poverty and homelessness) may also allow for intimate associations to quickly develop. The fact that the new setting is read by many of them as "hostile" and incongruent to what they are used to may also play a role in the almost spontaneous formation of quasi-familial relationships among these individuals. Their migrant habitus may be reinforced by current structural (socio-economic) limitations. Continued risky drug use and sexual behavior despite ample access to services in NYC seems to be the result of the combination of PR IDU-specific cultural demeanors with NYC-specific material barriers.

While the ARIBBA study [4] found that 41% of migrants moved to NYC to be with family and 7% to access drug treatment services, in our sample, 67% of recent migrants and 46% of the non-recent migrants reported the latter as a reason for migrating, with only 8% of recent migrants reporting migration to be with family. Recent migrants interviewed during formative research were recruited by churches in Puerto Rico that connected them with faith-based "drug treatment programs" in NYC which, for many reasons, they left. Their subsequent homelessness helps explain the elevated degree of material instability they experienced while in NYC: recent migrants had significantly higher levels of past year homelessness and poverty compared with both non-recent migrants and US-born IDU.

One social psychologist who studies PR IDU migration issues explained that it is still unclear how many PR IDU faith-based organizations bring each year to NYC [personal communication, Rafael Torruella, Ph.D., December 2010]. Regarding the influence of this type of drug treatment program over PR IDU migration into the United States he said, "It seemed that some local governments in the island were experimenting with relocating some of their most problematic drug users to some service agencies willing to provide them with services on the state-side. More recently, the relocation of these individuals is less of an emerging policy/experimentation and is becoming a more formal structure resulting from policy decisions" [32]. Although there are no written governmental policies that delineate this type of action, faith-based treatment programs seem to be a growing option for many in the island. However, it is still unclear what lasting impact the increasing religious currents among Puerto Rican policymakers and healthcare practitioners will have on the migration of IDU to NYC [11].

The location where IDU first start injecting drugs seems to play an important role in the development of cultural norms ingrained in these individuals' bodies and sense of "self" regulating their behavioral risk factors. The highest levels of syringe sharing were observed among the recent migrants, all but one of whom first injected in PR, while there were lower levels of sharing among non-recent migrants, a third of whom started injecting in NYC. In our study, how recently participants had made a residential move made an important difference in migrants' risk-taking behaviors. A certain kind of "mentality" nascent of a setting characterized by lack of syringe access continues to regulate these individuals' injection practices in NYC. This finding is further confirmed by a recent qualitative study that involved 24 in-depth interviews with PR IDU living in NYC revealing that mere access to free sterile injection supplies does not suffice to counteract risky injection behaviors that are largely explained by PR-specific cultural habits [personal communication, Yesenia Aponte-Meléndez, MA, May 2010]. This finding may suggest that learned risk-taking behaviors may take time and culturally-specific (PR IDU) risk prevention and education efforts to undo.

### Limitations

Since this is a cross-sectional study, we must exercise caution in attributing differences in risk to the migration experience. However, because this analysis mixed quantitative and qualitative research methods, the interpretation of our findings is very comprehensive. Also, by using RDS, we were able to access hidden populations within the overall IDU community in NYC and we were also able to obtain weighted estimates that potentially reduce the impact of peer recruitment bias on population estimates [30]. Finally, there is great uncertainty regarding the impact (if any) of faith-based drug treatment programs on the PR IDU migration phenomenon. Our findings concerning this phenomenon may not be generalizable to all PR IDU migrants in NYC.

## Conclusions

Puerto Rican migrants comprise a substantial portion of the NYC IDU population, and more IDU continue to migrate through faith-based and other programs. Because of the cultural norms of syringe sharing and risky sex that many migrant PR IDU bring, they now represent a particularly high-risk subpopulation of IDU within NYC. Despite increased HIV prevention and drug treatment services available in NYC, these migrants' drug and sexual risk behaviors are not being adequately addressed. While several HIV prevention programs, especially syringe exchange programs, provide many of these individuals with free and sterile injection equipment and condoms, access to injection equipment is not enough to address deeply-ingrained drug-use attitudes and practices. Thus far, one NYC syringe exchange program has included in its service portfolio an educational intervention that begins to address some of the recent IDU migrant-specific risk behaviors we have identified in this analysis. Our findings suggest that such deeply embedded risky practices require culturally appropriate prevention and education efforts that take into account the impact of the migration process (including poverty, homelessness and cultural marginalization), and the cultural norms many PR IDU bring to their new setting. Finally, unstable material conditions stemming from unexpected homelessness, (and, in our sample, resulting from faith-based programs' interventions in PR and in NYC), along with cultural barriers (i.e., language, different drug subcultures, etc.) converge to place these individuals in particularly risky situations. However, more research is needed to improve our understanding of the particularities of this PR IDU migration phenomenon. Improved drug treatment service provision and public health policies may result from such endeavor.

## Competing interests

The authors declare that they have no competing interests.

## Authors' contributions

CGA identified the research problem, contributed in the conceptual design and conducted all qualitative research and analysis included in this manuscript. HH contributed to the conceptual design, statistical analysis and overall writing, organization and development of this manuscript. SJ contributed to the statistical analysis and overall writing, organization and development of this manuscript. AN contributed to the editing and organization of the manuscript. TW contributed to the editing of the manuscript and provided important feedback on the qualitative analysis of this manuscript. All authors read and approved the final manuscript.

## Author's information

**CGA **holds a MA in Sociology from CUNY and is currently a PhD candidate at the New School for Social Research. Her dissertation explores poor heroin users' experiences with the disease model of active heroin use. She was the Project Director and Ethnographer of the NHBS study from 2008 to 2011. She is also Board Chair of "El Punto en la Montaña", a Syringe Exchange Program in rural Puerto Rico.

**HH**, PhD is an infectious disease epidemiologist and Director of the Interdisciplinary Research Methods Core of the Center for Drug Use and HIV Research at New York University. Her research has focused on the epidemiology and prevention of infectious disease consequences of illicit drug use. She is a member of the IOM Committee on the Prevention and Control of Viral Hepatitis in the United States.

**SJ **is a PhD student in the Department of Epidemiology at the University of Washington. At the time of this study he was a Research Scientist with the HIV Epidemiology Program at the New York City Department of Health and Mental Hygiene. His current research focuses on the social and structural determinants of heterosexual HIV risk and the analytic methods for estimating population characteristics of hard-to-reach groups.

**TW**, JD, PhD is a Research Associate and Scholar-In-Residence in the Department of Anthropology, John Jay College of Criminal Justice, City University of New York. He has been an ethnographer working with New York City drug users and distributors since 1996. His current activities include serving as Principal Investigator of the New York City National HIV Behavioral Surveillance, and a study of the repeal of the Rockefeller drug laws in New York State. His research interests center around the social organization of the distribution and consumption of illegal commodities, and the role of social networks in those processes. His favorite color is green.

**AN**, PhD is Director of Research in the HIV Epidemiology Program at the New York City Department of Health and Mental Hygiene. Since 1988, he has conducted research on the behavioral and social network risks for HIV/AIDS, viral hepatitis, and sexually transmitted infections among drug users and other at-risk populations in New York City, Newark, NJ, and in other locations.
